# Short Dark Tetrad - Polish version (SD4-PL): adaptation and extended psychometric evaluation of a measure of subclinical psychopathy, narcissism, Machiavellianism, and sadism

**DOI:** 10.3389/fpsyt.2026.1865917

**Published:** 2026-07-30

**Authors:** Paweł Dębski, Piotr Pałczyński, Monika Garczarczyk, Martyna Piankowska, Karolina Haratyk, Michał Meisner

**Affiliations:** 1 Clinical Department of Psychiatry, Faculty of Medical Sciences in Zabrze, Medical University of Silesia, Katowice, Poland; 2 Institute of Psychology, Faculty of Psychology, Humanitas University, Sosnowiec, Poland

**Keywords:** antagonistic traits, bifactor model, CAST, Machiavellianism, narcissism, Polish adaptation, psychopathy, sadism

## Abstract

The Short Dark Tetrad scale (SD4) is a brief self-report instrument designed to assess four socially aversive or antagonistic personality traits: subclinical Machiavellianism, narcissism, psychopathy, and sadism. The purpose of the present study was to provide an extended psychometric evaluation of the Polish version of SD4 (SD4-PL). Following translation and cultural adaptation of the 28 original items, data were collected using an online survey. After data screening, the final analytic sample comprised 931 Polish adults (mean age = 34.64 years, SD = 10.98; 76.8% women). To provide a comprehensive psychometric evaluation, the validation strategy combined split-sample exploratory and confirmatory factor analyses, ordinal CFA with WLSMV estimation, comparisons of one-factor, correlated four-factor, higher-order, bifactor, and merged-factor models, item-level diagnostics, internal consistency coefficients, convergent and discriminant validity indices, direct validation of the sadism subscale with the Comprehensive Assessment of Sadistic Tendencies (CAST), and an exploratory assessment of gender invariance. The correlated four-factor model showed partially acceptable fit in the full sample (CFI = .926, TLI = .919, RMSEA = .082, SRMR = .085) and was broadly replicated in the holdout sample. The bifactor model provided the strongest fit (CFI = .959, TLI = .952, RMSEA = .063, SRMR = .067), suggesting that SD4-PL scores reflect both specific dark/antagonistic traits and a broader common antagonistic core. Reliability was acceptable for narcissism, psychopathy, sadism, and the total score, although reliability for Machiavellianism was weaker (alpha = .67, omega = .68). The sadism subscale showed strong convergent validity with the CAST total score (r = .661). The SD4-PL may therefore be useful as a brief measure of dark/antagonistic traits in Polish-speaking adults, but interpretation of individual subscales, especially Machiavellianism and gender comparisons, should remain cautious.

## Introduction

Contemporary personality psychology has increasingly focused on socially aversive, antagonistic, and self-centered dispositions that may underlie exploitative, hostile, callous, or norm-violating interpersonal behavior. These traits are typically examined at the subclinical level, where they can shape everyday interpersonal functioning without necessarily implying a psychiatric diagnosis or a need for clinical intervention. The present study follows this subclinical and dimensional perspective, while also recognizing that the behaviors and attitudes captured by such scales may be relevant to clinical, forensic, and health-related research contexts (1).

In 2002, Paulhus and Williams proposed a collective account of three dispositional personality traits: Machiavellianism, narcissism, and psychopathy. Despite theoretical differences, systematic empirical associations between these traits became apparent, and they were collectively referred to as the Dark Triad of personality. Machiavellian individuals tend to show a strategic, cynical and manipulative interpersonal orientation, emotional coldness in social relationships, instrumental treatment of others, and weak attachment to conventional moral norms. Narcissism is typically associated with self-centeredness, entitlement, grandiosity, dominance, and a desire for admiration. Psychopathy is commonly associated with low fear or anxiety, deficient empathy and remorse, shallow affect, impulsivity, callousness, and norm-violating or aggressive tendencies (1–7). Narcissism can be further differentiated into grandiose and vulnerable forms, a distinction especially relevant when interpreting brief measures that primarily capture grandiose self-enhancement (5).

The term “dark” remains widely used because it is embedded in the names of established constructs and instruments such as the Dark Triad, Dirty Dozen, and Short Dark Tetrad. Nevertheless, recent conceptual discussion has argued that the label may be sensationalistic or stigmatizing and that terms such as antagonistic, aversive, or self-centered traits may describe the construct domain with greater precision. In the present article, the word “dark” is retained when referring to the established construct family and to the name of the SD4, whereas the broader interpretation of results is framed in terms of antagonistic and socially aversive personality characteristics (8).

The Dark Triad describes socially aversive personality traits of a subclinical nature. Although such traits may predispose individuals to hostile, exploitative, or manipulative behavior, their manifestations do not necessarily indicate clinical impairment. Individuals high on these traits may function adequately in many contexts and, under some circumstances, may even confer apparent adaptive advantages, including pragmatic decision making, social assertiveness, leadership-related behaviors, or psychological resilience (9–11). At the same time, the empirical overlap among the dark traits is substantial. Particularly strong associations are often observed between Machiavellianism and psychopathy, and all three traits have been related to broader constructs such as social exploitativeness, meanness, antagonism, and the general D factor of personality (12–14).

Several instruments have been developed to measure dark traits either separately or in brief aggregate forms. Longer measures, such as the Mach IV, Narcissistic Personality Inventory, and psychopathy inventories, provide detailed coverage of the construct but impose substantial time and cognitive demands when administered together. Brief multi-trait instruments, including the Short Dark Triad and the Dirty Dozen, were developed partly for reasons of measurement economy. Their brevity facilitates large-scale survey research and comparative studies, but it also increases the psychometric burden placed on individual items and may reduce precision at the subscale level (2, 15–17).

The Dark Triad has been expanded by including additional traits that meet the criterion of interpersonal antagonism or meanness. Special attention has been paid to sadism because the original triad does not fully explain the tendency to enjoy, seek, or be entertained by the suffering of others. Sadism is commonly defined as the tendency to derive pleasure from observing or causing physical or psychological suffering. Contemporary research focuses not only on sexual sadism but also on everyday or subclinical sadism, which may be expressed through enjoyment of cruelty, humiliation, violent entertainment, or interpersonal dominance in ordinary contexts (18–26).

The original self-report Short Dark Tetrad (SD4) was developed by Paulhus, Buckels, Trapnell, and Jones to measure four traits - Machiavellianism, narcissism, psychopathy, and sadism - using 28 items, with seven items assigned to each trait. The SD4 was created as an extension of shorter Dark Triad measures and was intended to provide a brief but broad screening tool for dark personality traits. It measures grandiose rather than vulnerable narcissism and provides a general assessment of psychopathy, Machiavellianism, and sadism rather than differentiating between all theoretically possible subtypes. Thus, the SD4 is best understood as a concise research measure rather than a comprehensive clinical instrument (15, 27–29). This scope is consistent with the original SD4 validation and subsequent work examining SD4 structure and correlates (27, 28).

Previous validation studies and adaptations have generally supported the practical usefulness of the SD4, but they have also revealed psychometric limitations. Several adaptations reported acceptable internal consistency but imperfect model fit, weaker items, and significant overlap among the subscales. These findings suggest that difficulties in obtaining ideal fit may not be limited to a single language version but may reflect broader challenges inherent to brief measurement of adjacent antagonistic constructs. In particular, the SD4 may capture both specific dark traits and a broader common antagonistic core (27–36).

The measurement of these traits is psychometrically challenging for several reasons. First, the constructs are conceptually adjacent and often substantially correlated, which makes it difficult to demonstrate discriminant validity, especially in short instruments. Second, the content of socially aversive items may produce skewed response distributions and floor effects because respondents are typically reluctant to endorse explicitly cruel, aggressive, or norm-violating statements. Third, short subscales must balance content coverage with brevity, so individual items may have a disproportionate influence on reliability, factor loadings, and model fit. These issues are particularly important in cross-cultural adaptations, where literal translation alone does not guarantee that item content, social acceptability, and construct boundaries operate in the same way as in the original version (35–37).

Polish psychology currently lacks a widely available, comprehensive instrument for measuring the four Dark Tetrad traits within a single brief questionnaire. A Polish adaptation of the SD4 may therefore support research on aversive and antagonistic personality traits, facilitate comparison with international findings, and provide a practical screening instrument for personality, clinical, and forensic psychology research. However, because cultural adaptation may introduce semantic, cultural, and response-style differences, validation should not be limited to a single confirmatory model. It should include exploratory and confirmatory structural analyses, item-level diagnostics, reliability estimates, convergent and discriminant validity, and, where possible, measurement invariance across relevant groups (27, 37).

For these reasons, validation of the SD4-PL requires more than simply reproducing the original four-factor structure. A stronger psychometric evaluation should examine whether the intended structure emerges empirically, whether it can be replicated in an independent subsample, whether the ordinal nature of the items is handled appropriately, and whether the four subscales can be distinguished from each other despite their common antagonistic content. It is also important to test whether sadism, the defining extension from the Dark Triad to the Dark Tetrad, shows direct convergence with an external sadism measure rather than only with measures of psychopathy or Machiavellianism (21, 27, 37). Against this background, the present study was designed to address these requirements.

## Aim

The purpose of the present study was to provide an extended psychometric evaluation of the Polish version of the Short Dark Tetrad scale (SD4-PL). In line with contemporary standards for test adaptation, the evaluation focused on: (a) exploratory and confirmatory evaluation of the factor structure using split-sample procedures; (b) ordinal CFA with an estimator appropriate for Likert-type items; (c) comparison of the original four-factor model with theoretically plausible alternatives, including one-factor, higher-order, bifactor, and merged-factor models; (d) reliability and item-level diagnostics; (e) convergent validity, including direct validation of the sadism subscale using CAST; (f) discriminant validity using latent correlations, HTMT ratios, AVE/CR criteria, and model comparisons; and (g) exploratory assessment of measurement invariance across women and men (37).

## Procedure for preparing the SD4-PL

The adaptation of the SD4 was carried out with the aim of enabling reliable psychometric evaluation in the Polish context. The goal of the process was to preserve content and construct equivalence between the original English-language version and the Polish version while maintaining the original 28-item, four-domain structure to allow comparison with the original SD4 and other language adaptations (27, 37).

The Polish version was prepared from the original items by two independent linguists specializing in translation and fluent in Polish and English, both experienced in translating psychological content. In addition, independent members of the research team - four psychologists and two psychology students fluent in Polish and English - prepared five additional Polish translations of the items. The final item wording was selected through consensus within the research team. The consensus procedure considered semantic equivalence to the original item, preservation of the target construct, linguistic clarity, naturalness in Polish, avoidance of unnecessary clinical or moralizing wording, and consistency with the response format. No formal independent back-translation and cognitive interviewing procedure was conducted; this is acknowledged in the limitations as a constraint of the adaptation process (37).

After the final version of the SD4-PL was prepared, a pilot phase was conducted to evaluate the clarity of instructions and items, the adequacy of the response scale, and potential interpretive difficulties. Participants were invited to report any ambiguities or concerns. No formal changes were made to the item content or procedure after the pilot phase; therefore, pilot responses were retained in the initial dataset before data screening (37). The original English SD4 items and the Polish SD4-PL wording are presented in **Table 1**.

**Table 1:** Original SD4 items and Polish SD4-PL wording.

Item	Trait	Original version	Polish version
SD4_01	M	It’s not wise to let people know your secrets.	Pozwalanie innym na poznanie twoich sekretów nie jest mądre.
SD4_02	M	Whatever it takes, you must get the important people on your side.	Należy za wszelką cenę mieć ważnych ludzi po swojej stronie.
SD4_03	M	Avoid direct conflict with others because they may be useful in the future.	Lepiej unikać bezpośrednich konfliktów z innymi, ponieważ w przyszłości mogą się oni przydać.
SD4_04	M	Keep a low profile if you want to get your way.	Jeśli chce się postawić na swoim, lepiej się nie wychylać.
SD4_05	M	Manipulating the situation takes planning.	Manipulacja sytuacją wymaga planowania.
SD4_06	M	Flattery is a good way to get people on your side.	Pochlebstwo jest dobrym sposobem na zjednanie sobie ludzi.
SD4_07	M	I love it when a tricky plan succeeds.	Cieszy mnie, gdy podstępny plan się powiedzie.
SD4_08	N	People see me as a natural leader.	Ludzie postrzegają mnie jako urodzonego lidera.
SD4_09	N	I have a unique talent for persuading people.	Mam wyjątkowy talent do przekonywania ludzi.
SD4_10	N	Group activities tend to be dull without me.	Beze mnie aktywności grupowe są zazwyczaj nudne.
SD4_11	N	I know that I am special because people keep telling me so.	Wiem, że jestem wyjątkowy(a), ponieważ ludzie ciągle mi to mówią.
SD4_12	N	I have some exceptional qualities.	Posiadam wyjątkowe cechy.
SD4_13	N	I’m likely to become a future star in some area.	Jest bardzo prawdopodobne, że w przyszłości zostanę gwiazdą w jakiejś dziedzinie.
SD4_14	N	I like to show off every now and then.	Lubię się popisywać od czasu do czasu.
SD4_15	P	People often say I’m out of control.	Ludzie często mówią, że nie panuję nad sobą.
SD4_16	P	I tend to fight against authorities and their rules.	Mam w zwyczaju walczyć przeciwko władzy oraz jej zasadom.
SD4_17	P	I’ve been in more fights than most people of my age and gender.	Uczestniczyłem(am) w większej liczbie bójek niż większość ludzi w moim wieku i mojej płci.
SD4_18	P	I tend to dive in, then ask questions later.	Zazwyczaj najpierw działam, potem myślę.
SD4_19	P	I’ve been in trouble with the law.	Miałem(am) problemy z prawem.
SD4_20	P	I sometimes get into dangerous situations.	Czasami pakuję się w niebezpieczne sytuacje.
SD4_21	P	People who mess with me always regret it.	Ludzie, którzy ze mną zadzierają, zawsze tego żałują.
SD4_22	S	Watching a fist-fight excites me.	Ekscytuje mnie oglądanie bójek.
SD4_23	S	I really enjoy violent films and video games.	Brutalne filmy i gry wideo sprawiają mi dużą przyjemność.
SD4_24	S	It’s funny when idiots fall flat on their face.	Bawi mnie, gdy idioci ponoszą druzgocącą porażkę.
SD4_25	S	I enjoy watching violent sports.	Lubię oglądać brutalne sporty.
SD4_26	S	Some people deserve to suffer.	Niektórzy ludzie zasługują na cierpienie.
SD4_27	S	Just for kicks, I’ve said mean things on social media.	Dla zabawy wypisuję złośliwe rzeczy w mediach społecznościowych.
SD4_28	S	I know how to hurt someone with words alone.	Wiem, jak kogoś zranić samymi słowami.

M, Machiavellianism; N, narcissism; P, psychopathy; S, sadism.

## Participants and data collection procedure

The online study was conducted among Polish-speaking participants recruited through social networks and other online channels using convenience sampling. The initial dataset contained 976 responses. During data screening, multivariate outliers were identified using the Mahalanobis distance (d²) calculated in IBM SPSS AMOS. A conservative cutoff point of p <.001 was applied. Based on this criterion, 41 observations were classified as multivariate outliers and excluded prior to the main psychometric analyses, corresponding to 4.2% of the initial dataset. The d² values for the excluded observations ranged from 119.972 to 59.492, and the first retained case had p = .001 and d² = 59.058, consistent with the predefined exclusion threshold. After this step, the dataset comprised 935 cases. Because the planned gender-related analyses, including measurement invariance testing, required two sufficiently large comparison groups, four participants who selected another gender category were not retained in the final analytic dataset. Consequently, the final analytic dataset comprised 931 adults aged 18–72 years (M = 34.64, SD = 10.98), including 715 women (76.8%) and 216 men (23.2%). The majority of participants had higher education (n = 558, 59.9%) or secondary education (n = 337, 36.2%), and the largest residential group lived in large cities of more than 100,000 inhabitants (n = 425, 45.6%).

All participants completed the questionnaires anonymously online. Before accessing the questionnaires, they received information about the purpose and duration of the study, the voluntary nature of participation, anonymity, and the right to withdraw at any time before submission. Participation proceeded only after electronic informed consent was indicated in the online form. The analyses reported in this manuscript are based exclusively on adult participants. The study was approved by the Bioethics Committee of the Medical University of Silesia in Katowice (BNW/NWN/0052/KB/236/24) and was conducted in accordance with the Declaration of Helsinki. The sociodemographic characteristics of the final analytic sample are presented in **Table 2**.

**Table 2:** Sociodemographic characteristics of the final analytic sample.

Characteristic	n/value	%
Final analytic sample	931	100.0
Women	715	76.8
Men	216	23.2
Age, M (SD), range	34.64 (10.98), 18-72	
Higher education	558	59.9
Secondary education	337	36.2
Vocational education	23	2.5
Primary education	13	1.4
Large city >100,000 inhabitants	425	45.6
Medium city 20,000-100,000	210	22.6
Small city <20,000	98	10.5
Village	198	21.3

Percentages are calculated relative to N = 931 unless otherwise stated.

## Measures

The study consisted of a one-time completion of a sociodemographic questionnaire, the SD4-PL, and established measures used to evaluate convergent and discriminant validity. The validation battery included instruments assessing Dark Triad traits, Machiavellianism, narcissism, psychopathy, and sadism, including CAST as a direct external criterion for the SD4-PL sadism subscale (16, 20, 27).

1. The Short Dark Tetrad (SD4), developed by Paulhus, Buckels, Trapnell, and Jones, was used in the Polish adaptation prepared by the research team (SD4-PL). The instrument contains 28 statements rated on a five-point response scale. In line with the original structure, seven items assess each of the four intended traits: Machiavellianism, narcissism, psychopathy, and sadism. Higher scores indicate a greater endorsement of the corresponding trait. In the present study, all 28 original items were retained to preserve comparability with the original SD4 and other language adaptations (27).

2. The Dirty Dozen Scale by Jonason and Webster, in the Polish adaptation by Czarna, Jonason, Dufner, and Kossowska, was used as a brief external measure of Dark Triad traits. The scale consists of 12 items rated on a five-point scale and provides subscale scores for Machiavellianism, narcissism, and psychopathy, as well as a total score reflecting overall Dark Triad intensity. Its inclusion allowed comparison between SD4-PL subscales and a widely used concise measure of the related triadic construct domain (16, 38).

3. The Mach IV questionnaire by Christie and Geis was used as an external measure of Machiavellianism. The construct emphasizes a manipulative interpersonal style, cynical beliefs about human nature, emotional detachment, pragmatic strategic orientation, and a weak attachment to conventional morality. The questionnaire consists of 20 items rated on a Likert-type scale. Polish versions of Mach IV have been used in previous studies and provide a theoretically appropriate criterion to evaluate the convergent validity of the SD4-PL Machiavellianism subscale (2, 39–42).

4. The Narcissistic Personality Inventory in the Polish adaptation (NARPI) was used as an external measure of narcissism. The instrument assesses narcissistic characteristics and subcomponents such as leadership, the need for admiration, self-sufficiency, and vanity. It also allows for a broader assessment of grandiose narcissistic tendencies, which are conceptually closest to the narcissism construct represented in the SD4. The use of NARPI therefore enabled evaluation of whether the SD4-PL narcissism scale converges most strongly with established narcissism indices rather than with non-corresponding dark traits (4, 43, 44).

5. Psychopathic traits were measured with the Polish adaptation of the Triarchic Psychopathy Measure (TriPM-41). The triarchic model conceptualizes psychopathy through three partially distinct components: boldness, disinhibition, and meanness. This structure is useful for validation because the SD4 psychopathy subscale is relatively short and does not attempt to represent all components of psychopathy in equal detail. Therefore, correlations with TriPM total and subscale scores provide information not only about general convergence but also about which components of psychopathy are most strongly represented in the SD4-PL (45–47).

6. The Comprehensive Assessment of Sadistic Tendencies (CAST) was used as a direct external validation measure for the SD4-PL sadism subscale. The CAST items assessed direct verbal sadism, vicarious sadism, and direct physical sadism. This measure was particularly important because direct validation of sadism is essential for a Dark Tetrad instrument; correlations of the SD4-PL sadism scale with psychopathy or Machiavellianism cannot substitute for convergence with an established sadism measure. In the present sample, the CAST total score showed high internal consistency (alpha = .82, omega = .84) (20).

## Statistical analysis

The analytic strategy was designed to provide a comprehensive psychometric evaluation of the SD4-PL. Data screening examined missingness, univariate distributions, and multivariate outliers. Mahalanobis distance was used to identify multivariate outliers among the SD4 item set; after outlier removal and retention of adult participants in two analyzable gender groups, the final dataset contained 931 complete SD4 protocols. Descriptive statistics included means, standard deviations, medians, ranges, skewness, and kurtosis. Given the ordinal response format and non-normal distributions of several items, CFA models were estimated primarily with the WLSMV estimator treating SD4 items as ordered categorical indicators. Maximum likelihood estimation was retained only as a sensitivity analysis and for comparison with the AMOS approach (48–51).

The sample was randomly divided into an exploratory subsample (n = 465) and a confirmatory holdout subsample (n = 466). Exploratory factor analysis (EFA) was conducted on the first subsample using polychoric correlations and oblimin rotation, which allows factors to correlate. Solutions with one to six factors were evaluated using fit indices, residuals, and interpretability. A parallel analysis based on observed and random eigenvalues was used as an additional empirical guide for factor retention. Confirmatory factor analysis (CFA) was then conducted on the holdout sample and the full sample (52, 53).

The following CFA models were compared: a one-factor model, the theoretically intended correlated four-factor model, a higher-order model, a bifactor model with a general dark/antagonistic factor and four specific factors, and two models in which theoretically overlapping traits were collapsed (psychopathy with sadism; Machiavellianism with sadism). The model fit was evaluated using CFI, TLI, RMSEA with 90% confidence intervals, and SRMR. The primary interpretation emphasized ordinal WLSMV results, whereas MLR and AMOS ML estimates were treated as sensitivity analyses (53–56).

As an additional model-refinement check, a limited set of residual covariances was examined. These covariances were selected on the basis of modification indices and substantive overlap between item pairs, including similarity in wording, proximity, or content. They were identified in the exploratory subsample and evaluated in the independent holdout subsample. The primary interpretation of factorial validity remained based on the unmodified structural models (53, 57, 58).

Reliability was evaluated using Cronbach’s alpha, standardized alpha, McDonald’s omega, and item-level diagnostics, including corrected item-total correlations, alpha if item deleted, and graded-response IRT discrimination parameters. Convergent validity with Pearson and Spearman correlations between SD4-PL scores and external measures of Machiavellianism, narcissism, psychopathy, Dark Triad traits, and sadism was examined. Discriminant validity was evaluated using latent factor correlations, HTMT ratios, AVE and composite reliability, Fornell-Larcker criteria, and comparisons with merged-factor models. Measurement invariance across women and men was examined using configural, metric, scalar, and strict models. WLSMV invariance models did not yield stable fit indices, apparently because sparse ordinal response patterns and the strong gender imbalance produced unstable estimation; therefore, MLR invariance models are reported as exploratory sensitivity analyses and interpreted cautiously (59–64).

## Results

### Data screening, subscale descriptives, and intercorrelations

The final analytic dataset contained 931 complete SD4-PL protocols. No missing SD4 data were observed. The final sample excluded 41 multivariate outliers and four participants from a gender category too small for the planned multigroup analyses. Several SD4 items showed notable positive skewness and floor effects, particularly those explicitly assessing antisocial or violent behaviors. The strongest floor effects occurred for SD4_17, SD4_19, SD4_22, SD4_23, SD4_25, and SD4_27, for which 72.1%-82.9% of participants selected the lowest response category. This distributional pattern supported the use of ordinal estimation in the primary CFA models.

As shown in **Table 3**, reliability was acceptable for narcissism, psychopathy, sadism, and the total SD4-PL score, whereas Machiavellianism remained marginally below the conventional.70 threshold. The intercorrelations among the SD4-PL subscales were positive and ranged from weak to moderate, with the strongest association observed between psychopathy and sadism (**Table 4**).

**Table 3:** Subscale descriptives and reliability of SD4-PL scores in the final sample.

Scale	Range	M	SD	Skew	Kurtosis	Alpha	Omega total
Machiavellianism	7-35	21.20	4.34	-.10	-.03	.671	.678
Narcissism	7-34	18.43	5.00	-.09	-.24	.799	.808
Psychopathy	7-31	13.67	4.32	.76	.41	.711	.722
Sadism	7-30	13.51	4.74	.74	.21	.754	.788
Total SD4	28-121	66.81	12.97	.31	.42	.845	.850

N = 931. Alpha, Cronbach’s alpha; omega, McDonald’s total omega. The Machiavellianism subscale remained slightly below the conventional.70 alpha threshold.

**Table 4:** Intercorrelations among SD4-PL subscales in the final sample.

SD4-PL subscale	Machiavellianism	Narcissism	Psychopathy	Sadism
Machiavellianism	(.671)			
Narcissism	.154***	(.799)		
Psychopathy	.263***	.342***	(.711)	
Sadism	.405***	.290***	.523***	(.754)

Pearson correlations are presented below the diagonal; Cronbach’s alpha coefficients are shown on the diagonal in parentheses. ***p <.001.

### Item-level diagnostics

The item-level analyses indicated that the weakest performance was concentrated in selected items rather than distributed evenly across the scale. In the WLSMV CFA, the lowest standardized loading was observed for SD4_01 (lambda = .294), followed by SD4_04 (lambda = .393), SD4_18 (lambda = .427), SD4_06 (lambda = .469), and SD4_05 (lambda = .498). The Machiavellianism subscale therefore remained the main source of psychometric weakness. The item-rest correlations and IRT discrimination parameters were consistent with this conclusion: SD4_01 showed both the lowest item-rest correlation and weak discrimination. All items were retained to preserve comparability with the original SD4, but the discussion explicitly recommends future evaluation or rewording of weaker items (27–29, 35, 36). Full item-level diagnostics, including standardized loadings, item-rest correlations, and IRT discrimination parameters, are provided in **Supplementary Table 1**.

### Exploratory factor analysis

In the exploratory subsample, fit improved systematically from one- to six-factor solutions. The four-factor solution showed RMSEA = .060, RMSR = .032, and TLI = .807, indicating a broadly interpretable but not ideal approximation to the theoretical SD4 structure. The five- and six-factor solutions showed better statistical fit, and the parallel analysis suggested retention of five components. Thus, EFA did not provide unequivocal evidence for a clean four-factor solution; instead, it suggested that the four intended domains are recoverable but that additional residual structure or construct heterogeneity may be present (52, 53). Although the parallel analysis suggested retention of five components, the four-factor solution was retained for CFA to maintain comparability with the theoretical SD4 structure and to test whether the intended four-domain model was recoverable despite this empirical recommendation. Detailed EFA fit indices are reported in **Supplementary Table 2**.

### Confirmatory factor analyses and model comparisons

The ordinal CFA results showed that the theoretically intended correlated four-factor model fit the data substantially better than the one-factor model (**Table 5**). In the full sample, the four-factor WLSMV model yielded CFI = .926, TLI = .919, RMSEA = .082, and SRMR = .085; the holdout sample showed a very similar pattern (CFI = .925, TLI = .918, RMSEA = .087, SRMR = .094). These results indicate that the intended four-factor structure is broadly recoverable, although its fit is not uniformly optimal.

**Table 5:** CFA model comparison and AMOS ML sensitivity analysis.

Model	Sample	Estimator	chi-square (df)	CFI	TLI	RMSEA [90% CI]	SRMR/RMR
One-factor	full	WLSMV	6165.92 (350)	.801	.785	.134 [.131,.137]	.126
Four-factor	full	WLSMV	2498.29 (344)	.926	.919	.082 [.079,.085]	.085
Four-factor	holdout	WLSMV	1562.14 (344)	.925	.918	.087 [.083,.092]	.094
Higher-order	full	WLSMV	2610.06 (346)	.923	.915	.084 [.081,.087]	.086
Bifactor	full	WLSMV	1512.05 (322)	.959	.952	.063 [.060,.066]	.067
Bifactor	holdout	WLSMV	951.05 (322)	.961	.955	.065 [.060,.070]	.075
P-S merged	full	WLSMV	2902.94 (347)	.913	.905	.089 [.086,.092]	.091
M-S merged	full	WLSMV	3148.03 (347)	.904	.896	.093 [.090,.096]	.095
AMOS modified four-factor with five residual covariances	full	ML	1596.18 (339)	.822	.802	.063 [.060,.066]	.098

Primary models were estimated with WLSMV and ordered SD4 items. The AMOS ML model is reported as a sensitivity/comparability analysis using the five content-justified residual covariances from the model-refinement analysis; AMOS reports RMR rather than SRMR.

The bifactor model provided the best overall fit in both the full and holdout samples. In the full sample, the bifactor WLSMV model yielded CFI = .959, TLI = .952, RMSEA = .063, and SRMR = .067. The holdout sample yielded comparable values (CFI = .961, TLI = .955, RMSEA = .065, SRMR = .075). This pattern indicates that SD4-PL responses are best understood as reflecting both specific traits and a broader common dark/antagonistic factor. Models collapsing psychopathy with sadism or Machiavellianism with sadism fit worse than the four-factor and bifactor models, suggesting that the overlap between these traits does not justify reducing them to a single combined factor (13, 14, 56).

**Figure 1:**
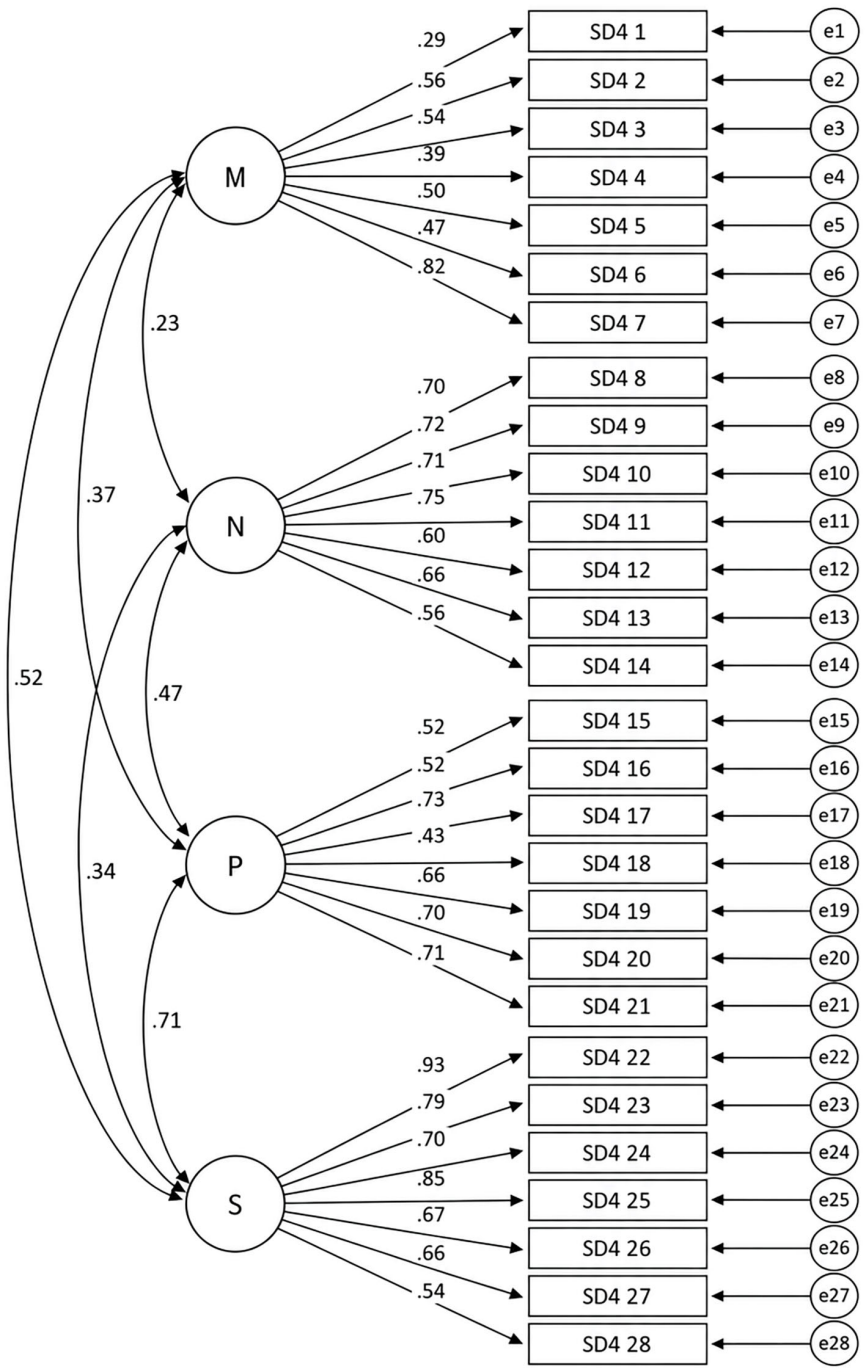
Standardized factor loadings and latent factor correlations in the primary correlated four-factor WLSMV CFA model of the SD4-PL without correlated residuals (N = 931). M, Machiavellianism; N, narcissism; P, psychopathy; S, sadism. Standardized coefficients are displayed. The model was estimated using WLSMV for ordered categorical items. Model fit: χ²(344) = 2498.29, CFI = .926, TLI = .919, RMSEA = .082, 90% CI [.079,.085], SRMR = .085.

As shown in **Figure 1**, all standardized factor loadings were positive and statistically significant, although their magnitude varied across subscales. The Machiavellianism items showed the weakest and most heterogeneous loadings, especially SD4_01 and SD4_04, whereas the narcissism and sadism items generally showed stronger loadings. The highest latent association was observed between psychopathy and sadism, whereas the weakest association was observed between Machiavellianism and narcissism. This pattern supports the recoverability of the intended four-factor structure but also indicates that the SD4-PL subscales should not be interpreted as fully independent or uniformly strong latent indicators.

### Residual covariances and sensitivity analysis

Residual covariances were estimated for five item pairs: SD4_08–SD4_09, SD4_03–SD4_04, SD4_15–SD4_18, SD4_11–SD4_12, and SD4_24–SD4_26. These pairs reflected overlapping wording or local content similarity, including leadership/persuasion, strategic conflict avoidance/low-profile self-presentation, impulsive dyscontrol, special/exceptional self-perception, and deserved failure/suffering. The modified model improved fit in the exploratory subsample (CFI from.928 to.951; RMSEA from.079 to.066) and also improved fit in the independent holdout sample (CFI from.925 to.941; RMSEA from.087 to.079). Thus, the residual correlations were not treated as purely *post hoc* modifications of a single sample; nevertheless, the unmodified four-factor and bifactor models remained the main basis for interpretation (57, 58). Detailed fit indices, including RMSEA confidence intervals, are provided in **Supplementary Table 3**.

### Discriminant validity

The latent correlations among the four WLSMV factors ranged from.234 to.714. The strongest association was observed between psychopathy and sadism (r = .714), consistent with their shared antisocial and aggressive content. HTMT ratios ranged from.303 to.769 and remained below conventional thresholds of.85 and.90, supporting acceptable discriminant validity. At the same time, the AVE values showed a more cautious picture: sadism exceeded.50 (AVE = .557), narcissism approached.50 (AVE = .455), whereas Machiavellianism (AVE = .284) and psychopathy (AVE = .385) were lower. The Fornell-Larcker criterion was most problematic for the psychopathy-sadism pair because the latent correlation exceeded the square root of AVE for psychopathy. These results support the distinguishability of the SD4-PL subscales at the HTMT level while confirming the significant overlap among the dark/antagonistic traits (62, 63). Because HTMT is more sensitive to discriminant-validity problems than the Fornell-Larcker criterion, the HTMT ratios were interpreted as the primary discriminant-validity index, with AVE/CR and Fornell-Larcker results used as complementary evidence (62, 63). Detailed AVE and composite-reliability values are presented in **Supplementary Table 4**. Latent correlations and HTMT ratios among SD4-PL factors are presented in **Table 6**.

**Table 6:** Latent correlations and HTMT ratios among SD4-PL factors.

Pair	Latent r	HTMT
M-N	.234	.303
M-P	.373	.357
M-S	.522	.505
N-P	.472	.471
N-S	.338	.375
P-S	.714	.769

HTMT values below.85/.90 are generally interpreted as supporting discriminant validity. The highest overlap was observed for psychopathy and sadism.

### Convergent validity

Convergent validity was evaluated by correlating the four SD4-PL subscales with external measures of corresponding and theoretically adjacent constructs (**Table 7**). The expected pattern was generally observed. SD4-PL Machiavellianism correlated most strongly with Mach IV and Dirty Dozen Machiavellianism; SD4-PL narcissism showed its strongest associations with NARPI total and leadership-related narcissism; SD4-PL psychopathy correlated most strongly with TriPM disinhibition and TriPM total; and SD4-PL sadism showed strong direct convergence with CAST total and vicarious sadism. Within the TriPM domains, the strongest association with disinhibition suggests that impulsive and poorly controlled behavioral tendencies are especially prominent in the SD4-PL operationalization of psychopathy. At the same time, several non-corresponding correlations were also moderate, reflecting the shared antagonistic core of the Dark Tetrad traits and reinforcing the need to interpret subscales as related but not fully independent constructs. The stronger association with CAST vicarious sadism is conceptually coherent because several SD4 sadism items emphasize enjoyment of violent or humiliating content and observation of suffering rather than direct physical aggression.

**Table 7:** Convergent validity correlations of SD4-PL subscales with external measures.

External criterion	Machiavellianism	Narcissism	Psychopathy	Sadism
DD-Psychopathy	.247***	.208***	.400***	.443***
DD-Narcissism	.381***	.453***	.282***	.373***
DD-Machiavellianism	.473***	.359***	.448***	.464***
DD-Overall result	.459***	.433***	.456***	.521***
TriPM-Boldness	-.028	.667***	.130***	.140***
TriPM-Disinhibition	.261***	.119***	.587***	.366***
TriPM-Meanness	.292***	.123***	.395***	.480***
TriPM-Overall result	.244***	.522***	.564***	.479***
MACH-Machiavellianism	.512***	.138***	.401***	.505***
NARPI-Leadership	.129***	.748***	.234***	.254***
NARPI-Admiration	.270***	.605***	.313***	.303***
NARPI-Self-Sufficiency	-.037	.564***	.115***	.059
NARPI-Vanity	.032	.538***	.158***	.131***
NARPI-Overall result	.151***	.750***	.265***	.253***
CAST-Direct verbal sadism	.275***	.211***	.368***	.413***
CAST-Vicarious sadism	.220***	.189***	.300***	.657***
CAST-Direct physical sadism	.254***	.222***	.436***	.488***
CAST-Overall result	.325***	.267***	.468***	.661***

Pearson correlations, N = 931. DD, Dirty Dozen; TriPM, Triarchic Psychopathy Measure; MACH, Mach IV; NARPI, Narcissistic Personality Inventory; CAST, Comprehensive Assessment of Sadistic Tendencies. ***p <.001. Spearman correlations showed the same substantive pattern and are recommended for supplementary reporting.

### Measurement invariance across gender

Because the sample was imbalanced by gender, measurement invariance was examined with caution. The WLSMV invariance models did not yield stable fit indices; therefore, MLR invariance models are reported only as exploratory sensitivity analyses. The MLR configural model converged but demonstrated poor overall model fit (CFI = .731, TLI = .704), well below the conventional.90 threshold, which limits strong conclusions about invariance. The decline in CFI from configural to metric and scalar models was small (Delta CFI = -.004 and -.009, respectively), whereas strict invariance showed a larger decrement (Delta CFI = -.048). These results do not provide strong evidence against metric or scalar equivalence, but the weak baseline fit and failed WLSMV invariance prevent firm claims that the SD4-PL is fully measurement invariant across women and men. Consequently, mean comparisons by gender should be interpreted with caution until invariance is confirmed in more balanced samples (64). Full exploratory MLR invariance fit indices are reported in **Supplementary Table 5**.

## Discussion

The present study provides an extended psychometric evaluation of the SD4-PL. The results support the utility of the Polish SD4 as a brief measure of dark/antagonistic personality traits, but also show that its latent structure should be interpreted with caution. The intended four-factor model was broadly recoverable and replicated in the holdout sample, yet its fit was not uniformly satisfactory. The bifactor model clearly provided the strongest fit, indicating that the SD4-PL captures both trait-specific variance and a broader common antagonistic core. This interpretation is consistent with theoretical accounts emphasizing a shared aversive or antagonistic factor underlying Machiavellianism, narcissism, psychopathy, and sadism (9, 13, 14, 27–29, 35, 36, 56).

The comparison of structural models was particularly informative. A one-factor solution was clearly inadequate, indicating that SD4-PL responses cannot be reduced to a single undifferentiated dark-trait score. At the same time, the bifactor model outperformed the correlated four-factor and higher-order models, suggesting that the general dark/antagonistic component is psychometrically salient. Models collapsing psychopathy with sadism or Machiavellianism with sadism fit worse than the four-factor and bifactor models. Thus, the overlap among traits is substantial but does not justify eliminating their distinct subscale interpretation (13, 14, 27–29, 56).

The pattern of fit indices also deserves methodological attention. The four-factor model showed an acceptable or near-acceptable absolute fit according to RMSEA and SRMR but weaker incremental fit indices, especially compared to more flexible models. Such discrepancies are not unusual in personality measurement, particularly when items have modest loadings, skewed ordinal distributions, and constructs share a common higher-order or bifactor structure. Therefore, the present results should not be interpreted solely through a single cutoff criterion. Rather, they illustrate the value of combining global fit indices, item-level diagnostics, alternative model comparisons, and external validity evidence when evaluating brief measures of complex antagonistic traits (49, 53–55, 58).

The item-level results help explain why the four-factor model did not achieve uniformly strong fit. Machiavellianism remained the most problematic subscale. Several Machiavellianism items showed lower standardized loadings or item-rest correlations, especially SD4_01. This pattern suggests that some items may capture pragmatic caution, privacy, or strategic self-presentation rather than the exploitative and manipulative core of Machiavellianism. Therefore, although the Machiavellianism subscale was retained to preserve comparability with the original SD4, future Polish-language research should consider cognitive interviewing, rewording, or alternative item formulations for this domain (27–29, 35, 36).

The present findings provide direct evidence for the convergent validity of the SD4-PL sadism subscale. Because sadism is the defining extension from the Dark Triad to the Dark Tetrad, validation against an external measure of sadistic tendencies is particularly important. The SD4-PL sadism subscale correlated strongly with the CAST total score, indicating that it captures variance directly related to sadistic tendencies rather than only variance shared with psychopathy, Machiavellianism, or broader Dark Triad measures. At the component level, the strongest association was observed with vicarious sadism. This pattern is conceptually coherent because several SD4 sadism items refer to the enjoyment of violent, humiliating, or cruel content rather than to direct physical aggression. Overall, these results support the interpretation of the SD4-PL sadism subscale as a valid indicator of everyday sadistic tendencies within the broader Dark Tetrad framework (20, 21, 25, 26).

The results of discriminant validity were mixed, but informative. HTMT ratios remained below the conventional thresholds, supporting the separability of the four subscales. However, the high latent correlation between psychopathy and sadism and the weaker AVE values for Machiavellianism and psychopathy indicate that the SD4-PL subscales should not be treated as fully independent constructs. Instead, the Polish SD4 appears to behave as a brief measure of interrelated antagonistic traits with both general and specific components. This interpretation is more consistent with the bifactor results than with the correlated four-factor model alone (13, 14, 56, 62, 63).

The exploratory gender invariance analyses require caution. Although the MLR models converged and the configural-to-metric and metric-to-scalar decrements were small, the configural model fit was weak and the WLSMV invariance models did not provide stable fit indices. Therefore, the present study cannot provide strong evidence for full measurement invariance across gender. This issue is important because the sample was strongly imbalanced by gender. Future research should test SD4-PL invariance in larger and more balanced samples and should consider partial invariance models or item-level differential functioning if non-invariance is detected (64).

From a practical perspective, the SD4-PL can be recommended as a brief research instrument to assess dark/antagonistic personality traits in Polish-speaking adults. The total score and the sadism and narcissism subscales appear particularly informative; the psychopathy subscale shows adequate reliability, whereas the Machiavellianism subscale should be interpreted with special caution due to weaker internal consistency, lower AVE, and several weaker item indicators. The SD4-PL should not be used as a clinical diagnostic instrument; rather, it is suitable for research screening, group-level analyses, and studies requiring a concise measure of aversive personality traits (27, 28, 36).

### Limitations

Several limitations should be acknowledged. First, the sample was recruited online using convenience sampling, with overrepresentation of women, individuals with higher education, and internet users. These characteristics restrict generalizability and may influence endorsement of socially aversive content. Second, although the final analytic sample included only adults and two analyzable gender groups, the gender distribution was strongly imbalanced, limiting the conclusiveness of the measurement invariance analyses. Third, the study did not include a measure of social desirability, impression management, or response style. This limitation is important because dark/antagonistic traits are socially undesirable and several SD4 items showed strong floor effects (8, 37).

Fourth, the translation procedure was based on multiple independent translations and expert consensus, but did not include formal independent back-translation or structured cognitive interviewing. This limits the strength of claims regarding full semantic equivalence. Fifth, although the bifactor model fits the data best, bifactor solutions can sometimes capitalize on item-level covariance and should be replicated in independent samples before strong theoretical conclusions are drawn. Sixth, WLSMV measurement invariance models did not provide stable fit indices, and the MLR invariance analysis should be treated as exploratory. Finally, the present study evaluated convergent validity using several established dark-trait instruments and CAST, but future research should extend the nomological network to normative personality models, especially Big Five agreeableness and conscientiousness, HEXACO honesty-humility, aggression, empathy, and behavioral criteria (12, 37, 56, 64). In particular, items with the weakest psychometric performance (SD4_01, SD4_04, and SD4_18) may benefit from future cognitive interviewing to determine whether their Polish wording adequately captures the intended Machiavellian or psychopathic content. Because no item changes were made after the pilot phase, pilot responses were included in the initial dataset before data screening; however, this means that the final analytic sample may include a small proportion of pilot respondents, which should be considered when interpreting the validation evidence.

## Conclusions

1. The Polish version of the Short Dark Tetrad (SD4-PL) shows acceptable overall psychometric utility as a brief measure of dark/antagonistic personality traits in adults, with reliability ranging from marginally acceptable for Machiavellianism to satisfactory for narcissism, psychopathy, sadism, and the total score.
2. The intended four-factor structure is broadly recoverable and replicable across a holdout sample, but the strongest structural support was obtained for a bifactor model, indicating the importance of a common dark/antagonistic core alongside specific traits.
3. The sadism subscale demonstrated strong direct convergent validity with CAST, particularly with CAST total and vicarious sadism, strengthening the validity evidence for the Dark Tetrad content of the SD4-PL.
4. Machiavellianism remains the weakest subscale and should be interpreted cautiously. Future studies should consider rewording, replacing, or further testing weaker items while preserving comparability with the original SD4 where necessary.
5. The SD4-PL may be used in research contexts as a concise measure of aversive personality traits, but gender comparisons and fine-grained interpretation of individual subscales should be approached cautiously until measurement invariance and differential item functioning (DIF) are more thoroughly investigated in larger and more balanced samples.

## Data Availability

The datasets presented in this study are openly available in the Open Science Framework (OSF) at: https://doi.org/10.17605/OSF.IO/YE9FU.
