# Supplementary material for: Short Dark Tetrad - Polish version (SD4-PL): adaptation and extended psychometric evaluation of a measure of subclinical psychopathy, narcissism, Machiavellianism, and sadism
Source: Front Psychiatry. 2026 Jul 30;17:1865917. doi: 10.3389/fpsyt.2026.1865917 (PMC13467914; doi:10.3389/fpsyt.2026.1865917)
Supplement: Supplementary file 1 [file Table1.docx]

# Supplementary material

**Supplementary Table S1. Full item-level diagnostics for SD4-PL items.**

| **Item** | **Trait** | **lambda** | **item-rest r** | **IRT a** |
| --- | --- | --- | --- | --- |
| SD4_01 | M | .294 | .227 | .616 |
| SD4_02 | M | .557 | .419 | 1.209 |
| SD4_03 | M | .539 | .509 | 1.871 |
| SD4_04 | M | .393 | .390 | 1.256 |
| SD4_05 | M | .498 | .372 | .976 |
| SD4_06 | M | .469 | .333 | .881 |
| SD4_07 | M | .822 | .423 | 1.157 |
| SD4_08 | N | .695 | .562 | 1.698 |
| SD4_09 | N | .722 | .560 | 1.598 |
| SD4_10 | N | .710 | .564 | 1.813 |
| SD4_11 | N | .750 | .629 | 2.254 |
| SD4_12 | N | .601 | .559 | 1.613 |
| SD4_13 | N | .661 | .534 | 1.600 |
| SD4_14 | N | .561 | .342 | .843 |
| SD4_15 | P | .522 | .440 | 1.111 |
| SD4_16 | P | .522 | .350 | .880 |
| SD4_17 | P | .735 | .432 | 1.828 |
| SD4_18 | P | .427 | .400 | 1.051 |
| SD4_19 | P | .661 | .393 | 1.762 |
| SD4_20 | P | .702 | .560 | 2.248 |
| SD4_21 | P | .705 | .425 | 1.258 |
| SD4_22 | S | .933 | .579 | 5.037 |
| SD4_23 | S | .793 | .564 | 3.306 |
| SD4_24 | S | .704 | .540 | 1.302 |
| SD4_25 | S | .850 | .526 | 3.652 |
| SD4_26 | S | .671 | .531 | 1.252 |
| SD4_27 | S | .660 | .368 | 1.448 |
| SD4_28 | S | .543 | .348 | .762 |

*Note. lambda = standardized factor loading from the correlated four-factor WLSMV model; item-rest r = corrected item-rest correlation; IRT a = graded response discrimination parameter.*

**Supplementary Table S2. Exploratory factor analysis fit indices for one- to six-factor solutions.**

| **Factors** | **RMSEA** | **TLI** | **RMSR** | **BIC** |
| --- | --- | --- | --- | --- |
| 1 | .101 | .450 | .072 | -127.6 |
| 2 | .085 | .610 | .054 | -569.0 |
| 3 | .072 | .722 | .039 | -813.8 |
| 4 | .060 | .807 | .032 | -946.2 |
| 5 | .054 | .842 | .027 | -937.1 |
| 6 | .045 | .891 | .023 | -944.9 |

*Note. EFA was conducted in the exploratory subsample (n = 465) using polychoric correlations and oblimin rotation. The parallel analysis retained five components by both mean and 95th-percentile random eigenvalue criteria.*

**Supplementary Table S3. Validation of selected residual covariances across exploratory and holdout subsamples.**

| **Model** | **Sample** | **CFI** | **TLI** | **RMSEA [90% CI]** | **SRMR** |
| --- | --- | --- | --- | --- | --- |
| Four-factor unmodified | EFA split | .928 | .921 | .079 [.074, .083] | .088 |
| Four-factor with selected residuals | EFA split | .951 | .945 | .066 [.061, .070] | .081 |
| Four-factor unmodified | holdout | .925 | .918 | .087 [.083, .092] | .094 |
| Four-factor with selected residuals | holdout | .941 | .934 | .079 [.074, .083] | .089 |

*Note. Residual covariances were selected in the exploratory subsample and tested in the holdout subsample. They are interpreted as local model refinements, not as primary evidence for construct validity.*

**Supplementary Table S4. AVE and composite reliability for the WLSMV four-factor model.**

| **Factor** | **AVE** | **CR** | **Minimum loading** | **Maximum loading** |
| --- | --- | --- | --- | --- |
| M | .284 | .718 | .294 | .822 |
| N | .455 | .853 | .561 | .750 |
| P | .385 | .809 | .427 | .735 |
| S | .557 | .895 | .543 | .933 |

*Note. AVE = average variance extracted; CR = composite reliability. AVE was strongest for sadism and weakest for Machiavellianism.*

**Supplementary Table S5. Exploratory MLR measurement invariance analysis across gender.**

| **Model** | **CFI** | **TLI** | **RMSEA** | **SRMR** | **Delta CFI** | **Delta RMSEA** |
| --- | --- | --- | --- | --- | --- | --- |
| configural | .731 | .704 | .075 | .084 |  |  |
| metric | .727 | .710 | .074 | .086 | -.004 | <.001 |
| scalar | .718 | .710 | .074 | .087 | -.009 | <.001 |
| strict | .670 | .673 | .079 | .092 | -.048 | .005 |

*Note. WLSMV invariance models did not return stable fit indices. MLR models are reported as sensitivity analyses. The configural model fit was weak; therefore, invariance conclusions are tentative.*
